# Supplementary material for: ECG-based beat-to-beat assessment of AV node conduction properties during AF
Source: Front Physiol. 2025 Aug 1;16:1624403. doi: 10.3389/fphys.2025.1624403 (PMC12354475; doi:10.3389/fphys.2025.1624403)
Supplement: Supplementary file 1 [file DataSheet1.pdf]

Supplementary Material

This supplementary material is divided into two sections. Section 1 describes the generation of the ground truth data used in the main article. Section 2 describes the model parameter ranges and the covariance matrix used for the propagation phase in the particle filter (PF) used in the main article.

1 GENERATION OF GROUND TRUTH DATA

Electrogram (EGM) recordings from the publicly available intracardiac atrial fibrillation database (iafdb) Goldberger et al. (2000) with the entire catheter resting against the atrial free wall were used to generate the simulated data. The RR and AA series were first extracted from this set of the iafdb dataset, as described in Section 2.2.1 in the main article. Further, the EGM-PF was run to estimate realistic model parameter trends, and the mode for each heartbeat was used as ground truth parameters, denoted $\hat{\boldsymbol{\theta}}^*$. The mode was calculated individually for each heartbeat k by sorting the M values into histogram bins with 5 ms width before identifying the center of the histogram bin with the highest count. These parameter trends together with the AA series from the iafdb recordings were further separated into minute-long segments with 25% overlaps to generate 25 realistic $\hat{\boldsymbol{\theta}}^*$ with associated AA series. To create augmented data segments, $\hat{\boldsymbol{\theta}}^*$ was time-reversed to create 25 additional minute-long segments. The 50 parameter trends $\boldsymbol{\theta}^*(k)$ with corresponding AA series were used to run the model to generate 50 simulated RR series, where the values for the four AV node properties (R^{FP}, R^{SP}, D^{FP}, and D^{SP}) were saved and used as ground truth, denoted $\boldsymbol{\phi}^*(k)$.

Moreover, the range between the highest and lowest values for each property in the simulations $\boldsymbol{\phi}^*(k)$ used for normalizing $\overline{l^1}$ to a percentage is $\boldsymbol{r} = [1450.9, 1050.5, 949.4, 1109.4]/100$ ms (division with 100 to convert the final normalized value to %).

2 PARTICLE FILTER DETAILS

The initialization of particles for the PF-EGM and PF-ECG is in the ranges seen in Table S1. These are also used as lower and upper limits for the AV node model parameters when running the PF. If a particle is moved outside of these boundaries due to the added noise in the propagation phase, the noise is redrawn until the particle is inside the boundaries. Furthermore, during the propagation phase, particles with an SP refractory period greater than the FP refractory period or an SP conduction delay less than the FP conduction delay are also redrawn.

The noise in the propagation phase follows a normal distribution with covariance $\Sigma = G \cdot \Sigma_{pre}$. The twelve AV node model parameters are known to interact with each other, and the covariance of this interaction, Σ_{pre}, was calculated based on all the estimated parameter vectors in a previously published article Karlsson et al. (2024) and is shown in Table S2. In that article, the same AV node model is used and the model parameters are estimated with a 10-minute resolution. This covariance lets the particles propagate in a purposeful direction, increasing the performance of the PF by reducing the number of particles needed. However, such a large added noise is not optimal for the PF, hence the scaling factor G is set to 0.3, chosen based on trial and error.

Table S1. Lower and upper boundaries for the initialization of particles for the PF-EGM and PF-ECG.

Model parameter	R^{FP}_{min}	ΔR^{FP}	τ^{FP}_{R}	R^{SP}_{min}	ΔR^{SP}	τ^{SP}_{R}	D^{FP}_{min}	ΔD^{FP}	τ^{FP}_{D}	D^{SP}_{min}	ΔD^{SP}	τ^{SP}_{D}
Lower boundary (ms)	100	0	25	100	0	25	2	0	25	2	0	25
Upper boundary (ms)	1000	1000	500	1000	1000	500	50	100	500	50	100	500

Table S2. Covariance Σ_{pre}.

Model parameter	R^{FP}_{min}	ΔR^{FP}	τ^{FP}_{R}	R^{SP}_{min}	ΔR^{SP}	τ^{SP}_{R}	D^{FP}_{min}	ΔD^{FP}	τ^{FP}_{D}	D^{SP}_{min}	ΔD^{SP}	τ^{SP}_{D}
R^{FP}_{min}	107883	-16980	3937	604	7530	3773	242	630	1236	132	-4	-1379
ΔR^{FP}	-16980	128708	-1547	769	11907	2768	20	1663	-1900	-118	-398	1418
τ^{FP}_{R}	3937	-1547	38065	-206	-273	2534	-21	-169	-263	-15	-86	-107
R^{SP}_{min}	604	769	-206	19137	-13678	-297	77	-199	-485	72	-1334	-1017
ΔR^{SP}	7530	11907	-273	-13678	54931	6222	-1	761	1546	-316	1903	4785
τ^{SP}_{R}	3773	2768	2534	-297	6222	33075	70	-7	156	70	333	-2725
D^{FP}_{min}	242	20	-21	77	-1	70	94	15	25	40	-9	-25
ΔD^{FP}	630	1663	-169	-199	761	-7	15	1103	-1254	24	141	316
τ^{FP}_{D}	1236	-1900	-263	-485	1546	156	25	-1254	40891	170	241	1230
D^{SP}_{min}	132	-118	-15	72	-316	70	40	24	170	288	-47	-157
ΔD^{SP}	-4	-398	-86	-1334	1903	333	-9	141	241	-47	1049	585
τ^{SP}_{D}	-1379	1418	-107	-1017	4785	-2725	-25	316	1230	-157	585	35940

REFERENCES

Goldberger, A. L., Amaral, L. A., Glass, L., Hausdorff, J. M., Ivanov, P. C., Mark, R. G., et al. (2000). Physiobank, physiotoolkit, and physionet: Components of a new research resource for complex physiologic signals. *Circulation [Online]* 101, e215–e220

Karlsson, M., Platonov, P. G., Ulimoen, S. R., Sandberg, F., and Wallman, M. (2024). Model-based estimation of av-nodal refractory period and conduction delay trends from ECG. *Frontiers in Physiology* 14, 1287365
